# Effects of aging and menopause on pancreatic fat fraction in healthy women population: A strobe-compliant article

**DOI:** 10.1097/MD.0000000000014451

**Published:** 2019-02-15

**Authors:** Wenjuan Yang, Yi Xie, Bin Song, Chunchao Xia, Chengwei Tang, Jing Li

**Affiliations:** a Department of Gastroenterology; b Department of Radiology, West China Hospital, Sichuan University, Chengdu, Sichuan, P.R. China.

**Keywords:** aging, fat, women, menopause, pancreas

## Abstract

Pancreatic fat fraction has been shown to increase in many pathological situations. However, pancreatic fat fraction and its physiological changes in healthy women are still unclear. The aim of this study is to investigate the effect of aging and menopause on pancreatic fat fraction in healthy female population.

This was a cross-sectional study. A phantom of fat–water mixtures was established. One hundred sixty-seven healthy women (20–70 years) were recruited. Fat fraction was quantified with double-echo chemical shift magnetic resonance imaging with T1 and T2∗ correction. The association between measured and actual fat fractions was determined with Pearson correlation. Linear regression analysis was used to establish the calibration curve. Fat fractions were analyzed via analysis of variance.

A significant positive linear correlation was revealed between the measured and actual fat fractions on the phantom (*r*^2^ = 0.991, *P* < .001). There was no significant difference in fat fractions among caput, corpus, and cauda of the pancreas. Pancreatic fat fraction remained constant during the age of 20 to 40 years (4.41 ± 0.79%) but significantly increased during the ages of 41 to 50 and 51 to 70 years (7.49 ± 1.10% and 9.43 ± 1.51%, respectively, *P* < .001). Moreover, pancreatic fat fractions of the healthy women aged 41 to 70 years were still significantly higher than these in the groups aged 20 to 40 years when postmenopausal healthy women were removed (*P* < .001). For volunteers aged 46 to 49 years, pancreatic fat fraction of the postmenopausal women was significantly increased compared with that of their premenopausal counterparts (*P* < .001).

We found that an even distribution of pancreatic fat in healthy women, aging and menopause as 2 independent risk factors for pancreatic steatosis, a fatty infiltration in the pancreas beginning in the fifth decade in women.

## 1 Introduction

The pancreatic fat content has been demonstrated to increase under various conditions. Focal fatty filtration in the pancreas has been reported in chronic pancreatitis, obesity, metabolic syndrome, steatohepatitis, cystic fibrosis, and pancreatic cancer, while diffuse pancreatic deposition has been observed in old age, non-alcoholic fatty liver disease, and alcoholic pancreatic steatosis.^[1–13]^ Ectopic fat accumulation in the pancreas may inhibit the secretion of pancreatic insulin and digestive enzymes,^[14,15]^ resulting in diabetes mellitus,^[15]^ steatorrhea,^[16]^ and malabsorption.^[17]^ Consequently, pathological pancreatic lipomatosis has received substantial attention.^[14–18]^ However, relatively few studies have investigated normal pancreatic fat content and distribution in a healthy population. In our previous study, fat content and an even fat distribution in healthy male pancreata were reported.^[19]^ Moreover, aging was shown as an independent risk factor for pancreatic steatosis in healthy male population.^[19]^ But these data from a healthy men population could not represent those of healthy women because sex hormones were found to act on the pancreas, and data regarding pancreatic fat fractions in healthy women may be different.^[20–22]^ Unfortunately, no studies clarifying the fat fraction and distribution in healthy women pancreata as well as the influence of age and menopause have been published.

Currently, double-echo chemical shift magnetic resonance imaging (CSI) is commonly used for noninvasive quantification of pancreatic fat content in clinical practice because of its quickness and convenience.^[23–25]^ Although the accuracy of double-echo CSI is susceptible to T1 and T2^∗^ relaxation effects, the influences could be minimized by the T1 and T2^∗^ correction.^[25]^ Several studies showed that there was a significant correlation between CSI and histopathological findings.^[2,19,26,27]^ Moreover, the accuracy of double-echo CSI was found comparable to that of magnetic resonance spectroscopy (MRS) after T1 and T2^∗^ correction.^[25]^ MRS is regarded as the reference standard for the noninvasive quantification of fatty liver.^[28]^ However, its time-consuming shimming and requirement for high homogeneity of the magnetic field make MRS inconvenient in routine clinical applications.^[19]^ Moreover, because the pancreas is much smaller and thinner than the liver, the region of interest (ROI) of the pancreas can not be set large enough, which make the accuracy of MRS is susceptible to the effects of respiratory interference and homogeneity of the magnetic field and limit its application in pancreatic fat quantification.^[19]^ In addition, Multi-Echo 3D GRE (gradient echo) and IDEAL-IQ (iterative decomposition of water and fat with echo asymmetry and least squares estimation quantification sequence) could compensate for T1 and T2^∗^ relaxation effects which would lower the accuracy of double-echo CSI.^[25]^ However, the clinical applications of Multi-Echo 3D GRE and IDEAL-IQ are still relatively less because they can only be used on certain magnetic resonance (MR) scanners with specific sequence.^[25]^

In the present study, we aimed to investigate the effects of aging and menopause on pancreatic fat fraction in healthy women population by using double-echo CSI with T1 and T2^∗^ correction.

## 2 Methods

### 2.1 Volunteers

This study was a cross-sectional study from January 2015 to July 2017 in West China Hospital of Sichuan University. This study was approved by the Chinese Clinical Trial Registry Clinical Trial Ethics Committee (registration number: ChiCTR-CCH-00000147) and the Ethics Committee of West China Hospital of Sichuan University. Informed consent was obtained from each participant prior to enrollment. One hundred sixty-seven healthy women volunteers aged 20 to 70 years were recruited into the study. The inclusion and exclusion criteria for the participants are listed in Table 1. The age distribution and menopausal status are shown in Table 2. Natural menopause was retrospectively diagnosed following 12 months of amenorrhea. The body mass index (BMI) was calculated as the weight (kg) divided by the square of the height (m^2^). The BMI of all participants was within the normal range (18–25 kg/m^2^).

**Table 1:**
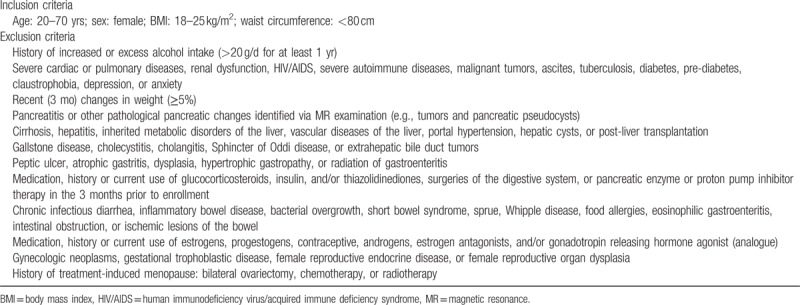
The inclusion and exclusion criteria for enrollment.

**Table 2:**
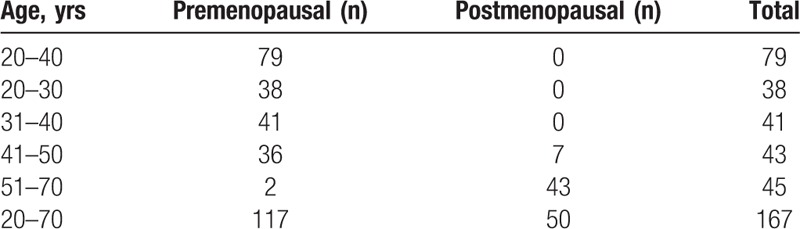
Participants distributed according to age and menopausal status (n = 167).

### 2.2 Construction of a fat–water mixture phantom

According to Namimoto et al,^[29]^ a fat–water mixture phantom was created with distilled water and 30% fat emulsion (intralipid, Sino-Swed Pharmaceutical Corp. Ltd., Beijing, China). The 30% intralipid was subsequently reconstituted to 15 dilutions (0%, 1%, 2%, 3%, 4%, 5%, 6%, 7%, 8%, 9%, 10%, 11%, 12%, 13%, and 14%) with distilled water in a 90 mL plastic vial, which was immersed in a water bath containing sodium chloride solution.

### 2.3 CSI technique

Imaging of the participants and the fat–water mixture was performed on a 3.0-T MR scanner (MAGNETOM Skyra, Siemens Healthcare, Erlangen, Germany) using double-echo CSI method with the parameters published by Yuan et al.^[25]^ In-phase (IP) and opposed-phase (OP) images were obtained via a T1-weighted two-dimensional spoiled double-echo gradient-echo sequence with the following parameters: repetition time (TR)/echo time (TE): 80/2.46 ms for IP images and 80/1.23 ms for OP images; flip angle: 50°; number of slices: 24; slice thickness: 5.0 mm; matrix size: 352 × 286; field of vision: 415 × 335 mm^2^; and scan time: 15 seconds in a single breathhold. Equal T2^∗^ of fat and water was estimated by a multi-echo spoiled gradient-echo sequence with the following parameters: TR: 9.15 ms; TEs: 2.46, 3.69, 4.92, 6.15, and 7.38 ms; flip angle: 4°; number of slices: 24; section thickness: 5 mm; matrix size: 160 × 95; field of vision: 420 × 315 mm^2^; and scan time: 13 seconds in a single breathhold. The volunteers underwent epigastric transverse scanning in the supine position. A respiratory belt was used to monitor the breathing cycle. All the patients received respiratory training before MR scan. All CSI studies were performed by an experienced technician. Slices were selected carefully to avoid large blood vessels, pancreatic duct, and peripancreatic fat as much as possible.

### 2.4 Image evaluation

All data were obtained and evaluated using the Siemens Syngo-imaging workstation. Images were independently reviewed in a randomized fashion by 2 radiologists with 5 and 10 years of experience, respectively. The radiologists were blinded to the clinical data. The signal intensity (SI) in the images was calculated with an operator-defined ROI in both the IP and OP images. For each phantom model, ROIs (80–100 mm^2^) were acquired from 3 adjacent sections (one ROI per section). For each volunteer, the ROIs (40–60 mm^2^) were drawn in the caput, corpus, and cauda of the pancreas (6 ROIs from 3 different layers per section) and were carefully placed to avoid the large blood vessels, pancreatic duct, and peripancreatic fat. The mean pixel SI values for each ROI were recorded using the vendor's software package. The fat fractions (FFs) were calculated with T1 and T2^∗^ corrected using the following formula (1) to (3)^[25,26,30]^:   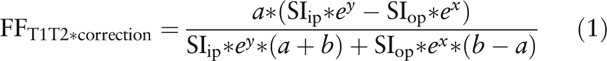



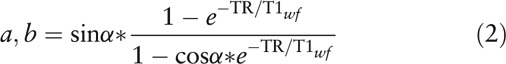



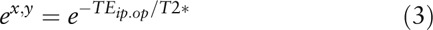


*S*_IP_ and *S*_OP_ indicate the SI in the IP and OP images, respectively. *α* indicates the excitation flip angle, *w* and *f* indicate water and fat, respectively.

The fat fractions of the caput, corpus, and cauda of the pancreas were averaged to determine the mean fat fraction of the whole pancreas.

### 2.5 Statistical analysis

All data were expressed as the means ± standard deviation (SD) and were analyzed using SPSS 13.0 software (SPSS, Chicago, IL). Pearson correlation was used to determine the association between the fat fractions measured with CSI and the actual value on the phantom. A linear regression analysis was used to establish the calibration curve. The pancreatic fat fraction data were analyzed via one-way analysis of variance followed by Dunnett T3 test. *P* < .05 was considered statistically significant.

The sample size was calculated according to the following formula^[19]^: 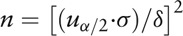


*σ* indicates the SD in the population. *δ* indicates the allowable error. According to the previously published data for healthy men (*σ* = 0.066, *δ* = 0.021), the sample size of each group should be at least 37 when *α* was set to 0.05.^[19]^

## 3 Results

### 3.1 Phantom study

**Figure 1:**
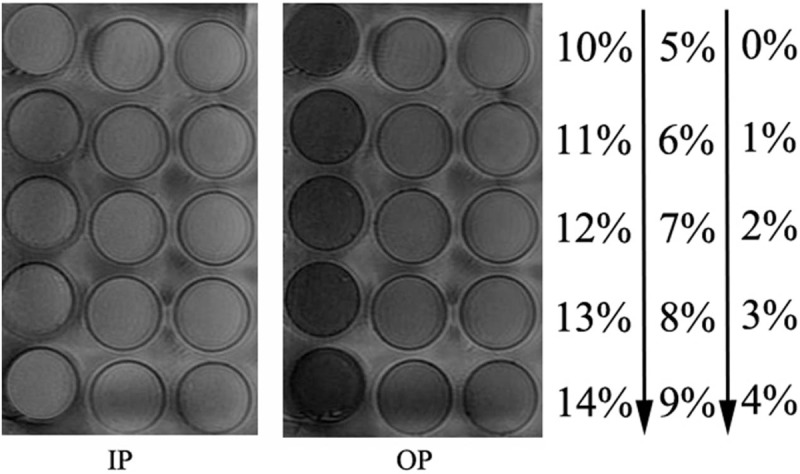
Chemical shift IP/OP MR images of the fat–water mixture phantom. The numbers on the right indicate the concentrations of the corresponding fat emulsions. IP = in-phase images; OP = opposed-phase images; MR = magnetic resonance.

**Figure 2:**
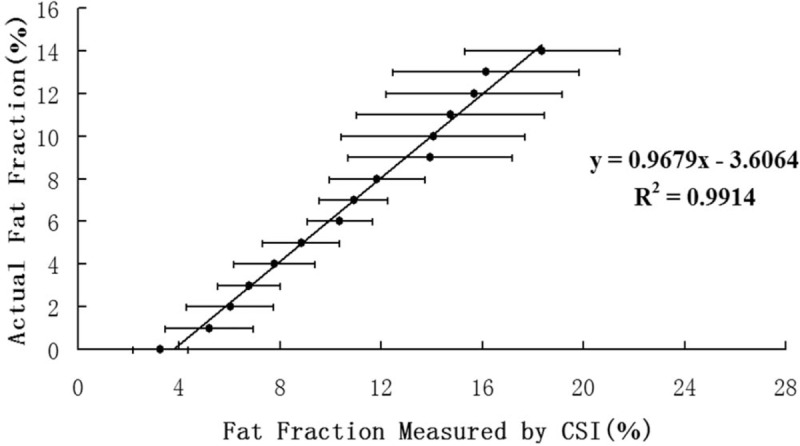
Scatter plots illustrate the correlation between the calculated and actual fat fractions. There is a positive linear correlation between the calculated and actual fat fractions. The calibration curve for the actual fat fractions originated in the linear regression equation.

The MR IP/OP images of the fat–water mixture phantoms were clear with an even SI (Fig. 1). There was a positive linear correlation between the calculated and actual fat fractions of the 15 fat–water standard samples (*R*^2^ = 0.991; *P* < .001) (Fig. 2). The linear regression equation (*P* < .001) between the actual 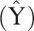 and calculated fat fractions 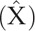 was acquired as follows: 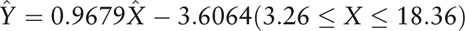


The regression equation was used as the calibration curve to estimate the actual fat fractions.

### 3.2 Even distribution of pancreatic fat in healthy women

**Figure 3:**
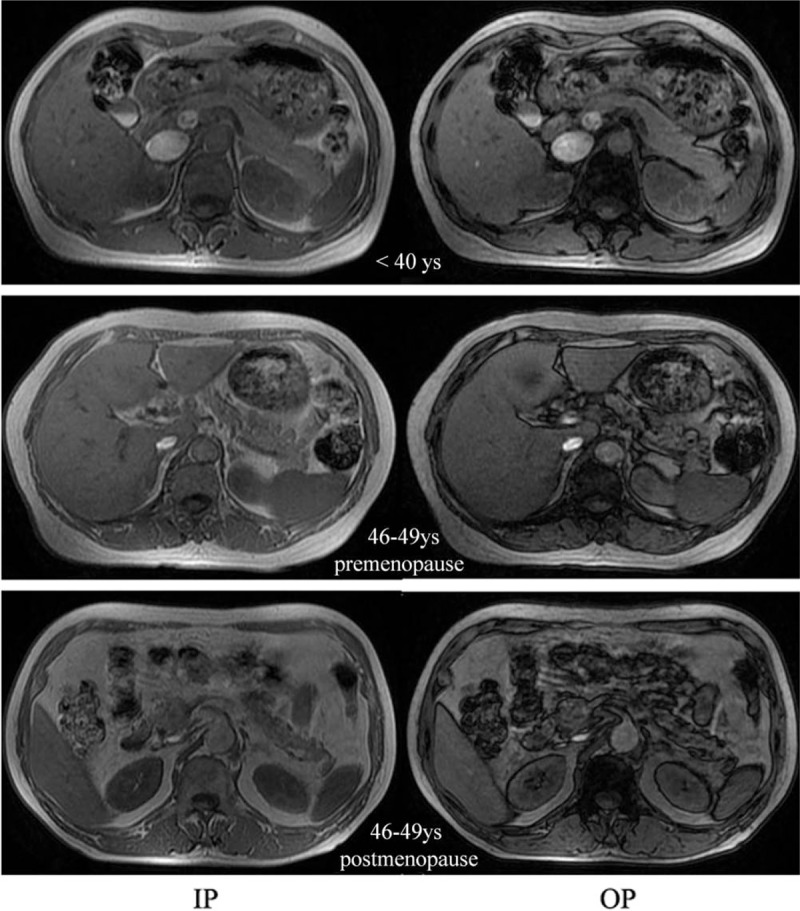
Chemical shift IP/OP MR images of the pancreas in healthy women. Pancreatic fat is indicated as a significant reduction in the signal intensity in the OP MR images compared with the IP MR images. The features of fatty infiltration in the pancreas begin in the fifth decade of healthy women and are more obvious in the women after menopause. IP = in-phase images; OP = opposed-phase images; MR = magnetic resonance; yrs = years.

As shown in Fig. 3, pancreatic fat was evenly distributed in the caput, corpus, and cauda of the pancreas. There was no significant difference in the fat fractions among the caput, corpus, and cauda of the pancreas in all age groups (*P* > .05) (Table 3).

**Table 3:**
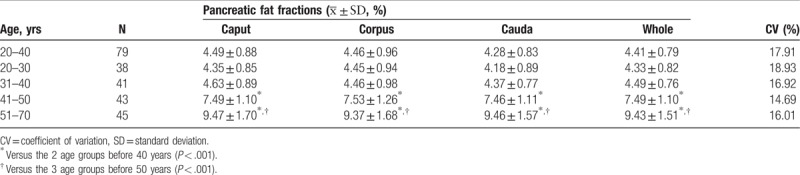
Pancreatic fat fractions in healthy women (20–70 years).

### 3.3 Effect of age on pancreatic fat fractions in healthy women

As listed in Table 3, there was no significant difference regarding the fat fractions in the 3 different pancreatic regions or the whole pancreas between the 20 to 30 and 31 to 40 years groups (*P* > .05). The mean value of the whole pancreatic fat fraction in the 20 to 40 years group was 4.41 ± 0.79% (95% confidence interval [CI] 4.23–4.59%; coefficient of variation [CV] = 17.91%). However, there were significant increases in the fat fractions of the 3 pancreatic regions and the whole pancreas in the 41 to 50 years group compared with the groups younger than 41 years (*P* < .001; Table 3). The mean value of the whole pancreatic fat fraction in the healthy women aged 41 to 50 years was 7.49 ± 1.10% (95% CI 7.15–7.83%, CV = 14.69%). And the fat fractions in the 3 pancreatic regions and the whole pancreas of the healthy women aged 51 to 70 years were dramatically increased compared with the groups aged 41 to 50, 31 to 40, and 20 to 30 years (*P* < .001; mean value 9.43 ± 1.51%; 95% CI 8.98–9.88%; CV = 16.01%). Moreover, the fat fractions in the 3 pancreatic regions and the whole pancreas of the healthy women aged 41 to 70 years were still significantly higher than these in the groups aged 20 to 30, 31 to 40, and 20 to 40 years when postmenopausal healthy women were removed (*P* < .001, Table 4).

**Table 4:**
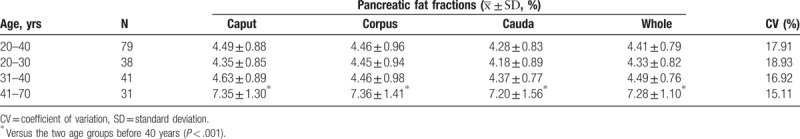
Pancreatic fat fractions in premenopausal women (20–70 years).

### 3.4 Effect of menopause on pancreatic fat fractions in healthy women

To determine whether menopause affects the pancreatic fat fraction, the women aged 46 to 49 years were chosen to make the ages of the premenopause and postmenopause women comparable. They were divided into 2 groups according to the presence or absence of menopause. There was no significant difference in age between the 2 groups. The fat fractions in the 3 pancreatic regions and the whole pancreas of the postmenopausal healthy women (mean value 9.03 ± 0.78%; 95% CI 8.19–9.86%; CV = 8.64%) were significantly increased compared with their premenopausal counterparts (mean value 7.25 ± 0.60%; 95% CI 6.92–7.59%; CV = 8.27%; *P* < .001; Table 5) (Fig. 3). Moreover, the pancreatic fat fraction of the premenopausal healthy women aged 46 to 49 years was still higher than that of the women aged 20 to 40 years (7.25 ± 0.60% vs 4.41 ± 0.79%, *P* < .001) (Fig. 3).

**Table 5:**
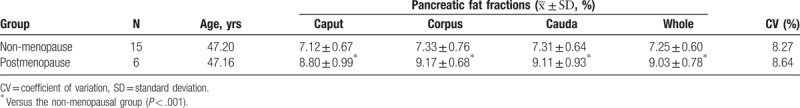
Pancreatic fat fractions in premenopausal and postmenopausal women (46–49 years).

## 4 Discussion

Compared with that of MRS, Multi-Echo 3D GRE, and IDEAL-IQ, the accuracy of double-echo CSI is more susceptible to T1 and T2^∗^ relaxation effects.^[25]^ But currently, considerable advances have been made in minimizing the influences by correcting the T1 and T2^∗^ relaxation using formulas (1) to (3) as described in the methods section. In addition, the used phantom is not a perfect representative for the pancreas mainly due to the different B1 homogeneity and the influence of the respiratory movement in patients. Thus, in order to reduce influence caused by breathing movements, an abdominal belt was used and all the patients received breath-hold training before MR scan. Moreover, the pancreas is a retroperitoneal organ, which is less affected by breathing movement during scanning than the intraperitoneal organs. Therefore, the phantom used in this study could reflect the tendency of fat contents of the pancreas related to the age of women. After correcting T1 and T2^∗^ relaxation, the actual fat fraction could be estimated by the calculated fat fraction and linear regression equation in the current and previous studies.^[19,25,30,31]^

In this study, we identified an even distribution of pancreatic fat in healthy women of all age groups. This result was consistent with our previous findings in healthy men aged 20 to 70 years.^[19]^ In addition, diffuse pancreatic steatosis has also been observed in obesity and non-alcoholic fatty liver disease.^[2,3]^ However, pancreatic focal fatty infiltration or replacement has frequently been reported in a variety of diseases, such as chronic pancreatitis, cystic fibrosis, pancreatic cancer, obesity, metabolic syndrome, and steatohepatitis.^[1–12]^ Nevertheless, we found that it did not appear in the pancreata of healthy women or men in the present study and our previous study.^[19]^ These findings suggest that focal fatty accumulation in the pancreas is closely related to the abovementioned states of patients and should be considered as a pancreatic pathologic change.^[4,6,9–11]^ Increasing evidence supports that pancreatic steatosis could impair pancreatic endocrine and exocrine functions.^[5,8,14,15]^ Whether related symptoms occur might depend on the severity of fatty accumulation.^[16,17]^ It was found that some patients were usually asymptomatic with mild fatty replacement, while others developed diabetes mellitus, diarrhea, or malabsorption with severe pancreatic lipomatosis.^[5,8,12–18]^ Thus, the quantitative detection of lipid overload in pancreatic tissue is important for identifying lipomatosis-induced disorders. In several studies, pancreatic steatosis was diagnosed when there was an increase in the ultrasonographic echogenicity of the pancreatic body compared with the kidney.^[10,11,32]^ However, the severity of pancreatic steatosis was not quantified in these studies. As the pancreas contains a certain amount of fat under physiological conditions, the premise of quantifying pancreatic steatosis is to establish the normal range of pancreatic fat fraction values and its physiological changes. Wong et al^[33]^ reported that 90% of healthy volunteers from the general population had pancreatic fat between 1.8% and 10.4%. However, that study was not controlled for sex or age. In recent years, our group has established a pancreatic fat fraction database of healthy men (2.8 ± 0.66% and 6.32 ± 1.18% for healthy men aged 20–50 and 51–70 years, respectively).^[19]^ The present study further demonstrated that the pancreatic fat fractions of healthy women aged 20 to 40, 41 to 50, and 51 to 70 years were 4.41 ± .79%, 7.49 ± 1.10%, and 9.43 ± 1.51%, respectively. The establishment of normal ranges of pancreatic fat fraction values in healthy populations would be helpful in the quantitative identification of pancreatic lipid overload.

In our studies, healthy women (20 to 40, 41 to 50, and 51 to 70 years) had a higher pancreatic fat fraction than their male counterparts of the same age.^[19]^ The underlying mechanism is still not clear. The pancreatic fat fraction was reported to be positively associated with the amount of visceral fat tissue.^[3,34]^ Interestingly, androgens were found to reduce fat body mass and inhibit abdominal fat accumulation in men.^[21,35,36]^ Consistent with these results, women (n = 131) were demonstrated to have increased fat mass (*P* < .001) compared with men (n = 103) among non-obese healthy volunteers in a randomized controlled clinical trial.^[37]^ These findings indicate that the difference in the pancreatic fat fraction between healthy women and men may be attributed to differences in body fat metabolism involving distinct sex hormones. Nevertheless, this hypothesis requires further experimental confirmation.

Several researchers have identified a positive correlation between age and the pancreatic fat fraction.^[38–40]^ That finding may be a result of the degeneration of pancreatic cells during aging.^[28,41,42]^ In addition, a gradual increase in oxidative stress during human aging may be responsible, in part, for the increased pancreatic fat fraction by triggering lipid droplet accumulation through activating fatty acid synthesis.^[43–45]^ However, it is unclear when pancreatic steatosis first occurs in healthy women. Computed tomography data from Saisho research demonstrated that pancreatic fat volumes remain remarkably unchanged throughout the second to ninth decades, but the pancreatic volume decreased after age 60 in women, which indicates that the pancreatic fat fraction may increase after age 60 in women.^[38]^ Nevertheless, the data from Saisho study could not accurately represent healthy women because individuals with obesity and diabetes mellitus were recruited. Using CSI, we found that the pancreatic fat fraction of healthy women with a normal BMI remained unchanged between 20 and 40 years of age; however, it significantly increased during the ages of 41 to 50 and 51 to 70 years by 70.0% and 25.9%, respectively. These findings suggested that the increase in the pancreatic fat fraction was initiated in the fifth decade.

According to the data from healthy men in our previous study, the increase in pancreatic fat fraction began in the 6 decade.^[19]^ We found that healthy women had fatty infiltration in the pancreas approximately 10 years earlier than healthy men. That result implied that factors in addition to aging affect the pancreatic fat fraction in women. Estradiol and progesterone have been demonstrated to protect the pancreas from lipomatosis by stimulating pancreatic cell proliferation,^[46]^ reducing pancreatic oxidative stress,^[20]^ and attenuating acinar cell apoptosis.^[21]^ In general, menopause occurs in women older than 40 years.^[47,48]^ The decrease in estradiol and progesterone levels after menopause diminished their protective effects on the pancreas and may have resulted in pancreatic fat infiltration. The findings of the present study support this hypothesis. We found that the pancreatic fat fraction of postmenopausal women was significantly higher than that of premenopausal women of the same age (46–49 years; 9.06 ± 0.73% vs 7.25 ± 0.60%, respectively). These findings suggest that menopause contributes to the earlier pancreatic fat deposition in healthy women. In addition, we found that the pancreatic fat fraction of premenopausal healthy women aged 41 to 70 years was still higher than that of women aged 20 to 40 years, which suggested that aging was an independent risk factor for pancreatic fat deposition in healthy women and that both aging and menopause were responsible for the increased pancreatic fat fraction of healthy women older than 40 years.

There are several limitations in this study. First, it is a single-center study. Second, there was no histological confirmation of the pancreatic fat fraction. Third, to make the ages of the premenopause and postmenopause women comparable, volunteers aged 46 to 49 years were chosen and the number of cases included in the analysis regarding the effect of menopause on the pancreatic fat fraction was relatively small. Fourth, the currently used imaging technique in this study is not ideal one because of the effect of T1 and T2^∗^ relaxation effects. Fifth, the used phantom is not a perfect representative for the pancreas mainly due to the different B1 homogeneity and the interference of the respiratory movement.

## 5 Conclusion

Using the double-echo CSI with T1 and T2^∗^ correction, we identified an even distribution of pancreatic fat in healthy women; pancreatic fat fractions of 4.41 ± 0.79%, 7.49 ± 1.10%, and 9.43 ± 1.51% in healthy women aged 20 to 40, 41 to 50, and 51 to 70 years, respectively; pancreatic steatosis beginning in the fifth decade among healthy women; and aging and menopause as 2 independent risk factors for pancreatic fat deposition in healthy women.

## Acknowledgments

The authors are grateful to the excellent statistical assistance from Prof. Jiayuan Li (West China School of Public Health, Sichuan University) and excellent technical assistance from Dr. Fang Yuan (Department of Radiology, West China Hospital, Sichuan University).

## Author contributions

**Conceptualization:** Chengwei Tang, Jing Li.

**Data curation:** Wenjuan Yang, Yi Xie, Bin Song.

**Formal analysis:** Wenjuan Yang.

**Funding acquisition:** Jing Li.

**Investigation:** Wenjuan Yang, Yi Xie.

**Methodology:** Wenjuan Yang, Yi Xie, Bin Song, Chunchao Xia.

**Project administration:** Jing Li.

**Software:** Wenjuan Yang.

**Supervision:** Jing Li, Chengwei Tang.

**Validation:** Jing Li.

**Writing – original draft:** Wenjuan Yang.

**Writing – review & editing:** Chengwei Tang, Jing Li.
